# Characterization of Disease-Associated Mutations in Human Transmembrane Proteins

**DOI:** 10.1371/journal.pone.0151760

**Published:** 2016-03-17

**Authors:** János Molnár, Gergely Szakács, Gábor E. Tusnády

**Affiliations:** Institute of Enzymology, Research Centre for Natural Sciences, Hungarian Academy of Sciences, H-1117, Budapest, Hungary; University of the Sunshine Coast, AUSTRALIA

## Abstract

Transmembrane protein coding genes are commonly associated with human diseases. We characterized disease causing mutations and natural polymorphisms in transmembrane proteins by mapping missense genetic variations from the UniProt database on the transmembrane protein topology listed in the Human Transmembrane Proteome database. We found characteristic differences in the spectrum of amino acid changes within transmembrane regions: in the case of disease associated mutations the non-polar to non-polar and non-polar to charged amino acid changes are equally frequent. In contrast, in the case of natural polymorphisms non-polar to charged amino acid changes are rare while non-polar to non-polar changes are common. The majority of disease associated mutations result in glycine to arginine and leucine to proline substitutions. Mutations to positively charged amino acids are more common in the center of the lipid bilayer, where they cause more severe structural and functional anomalies. Our analysis contributes to the better understanding of the effect of disease associated mutations in transmembrane proteins, which can help prioritize genetic variations in personal genomic investigations.

## Introduction

Completion of the Human Genome Project resulted in a significant progression in genetic research. The publication of the human reference sequence ignited several remarkable projects, such as the 1000 Genomes Project [1], which provided a comprehensive resource of human genetic variation; the Cancer Genome Atlas [2], which was launched to identify genetic mutations in distinct tumor types; or the ENCODE project [3], which was established to identify functional genomic elements. Despite the spate of data emerging from these projects, the relevance of individual variations is not fully understood [4]. Transmembrane proteins (TMPs) perform essential roles in cellular functions. Consequently, the smallest alteration in the sequence of these proteins can have severe or fatal [5–8] effect. Furthermore, these proteins participate in the communication between the cell and the environment, hence they can be potential targets of drugs. Analysis of genetic variations in the context of the 3D structure of TMPs may help efforts to distinguish disease causing mutations and natural polymorphisms. A notable example of this type of investigation was the mapping of disease associated mutations to the homology model of human *ABCC6*, which is responsible for pseudoxanthoma elasticum (PXE). In this study, significant clustering of the missense mutations was found at complex domain-domain interfaces: at the transmission interface that involves four intracellular loops and the two ABC domains as well as at the ABC-ABC interacting surfaces [9]. However, 3D structure determination of TMPs lags behind the structure determination of globular proteins since the crystallization of these proteins requires special techniques, and their size frequently limits investigations by NMR spectroscopy. Fortunately, low-level structural information such as the transmembrane topology of the proteins can be determined by various experimental techniques [10,11] and can be also predicted with high accuracy [12–15]. A previous study of 80 TMPs has shown that disease-causing glycine to arginine changes are statistically frequent in transmembrane (TM) regions [16]. There is strong evidence that these highly charged mutations can cause misfolding of TMPs, which is one of the reasons behind the dysfunction of these proteins [17]. In the case of *FGFR3*, the extra charge in the TM region provided by the arginine leads to a disease [18]. However, it was also shown that arginine can play a naturally essential role in the function of several TMPs, for example the voltage-gated potassium channel KvAP contains arginines in the S4 hydrophobic segment [18].

The Human Transmembrane Proteome (HTP) database is one of the most complete resources containing topology as well as 3D structural information of human TMPs [19]. This comprehensive database provides a unique opportunity to examine the distribution of missense genetic variations and the spectrum of amino acid substitutions across the topological segments of the human transmembrane proteome. In this work we analyzed the HTP to characterize disease causing mutations and polymorphisms in the context of transmembrane topology and KEGG enrichment.

## Results

### Genetic variations within transmembrane proteins

Genetic variations listed in the UniProt database [20] were mapped to the human transmembrane proteome. Altogether, 19513 genetic variations were identified, including 10952 polymorphisms and 8561 disease associated variants in 3153 and 642 TMPs, respectively (S1 File). This result shows that there are about five times more TMPs carrying polymorphisms than TMPs containing disease associated mutation(s). In the case of non-TMPs, we identified 26829 polymorphism and 15990 disease associated mutations within 8472 and 1552 proteins, respectively. The rate of polymorphisms is 8,76x10^-3^ and 4,56x10^-3^ per residue in the TMPs and non-TMPs, respectively. The rate of disease associated mutations is 2,57x10^-2^ and 1,36x10^-2^ per residue in the TMPs and non-TMPs, respectively. These data show the relative enrichment of disease associated mutations in TMPs, which may be explained by the reduced tolerance of TMPs to mutations.

### TMPs with genetic variations have biased distribution across categories of different TM region counts

**Fig 1:**
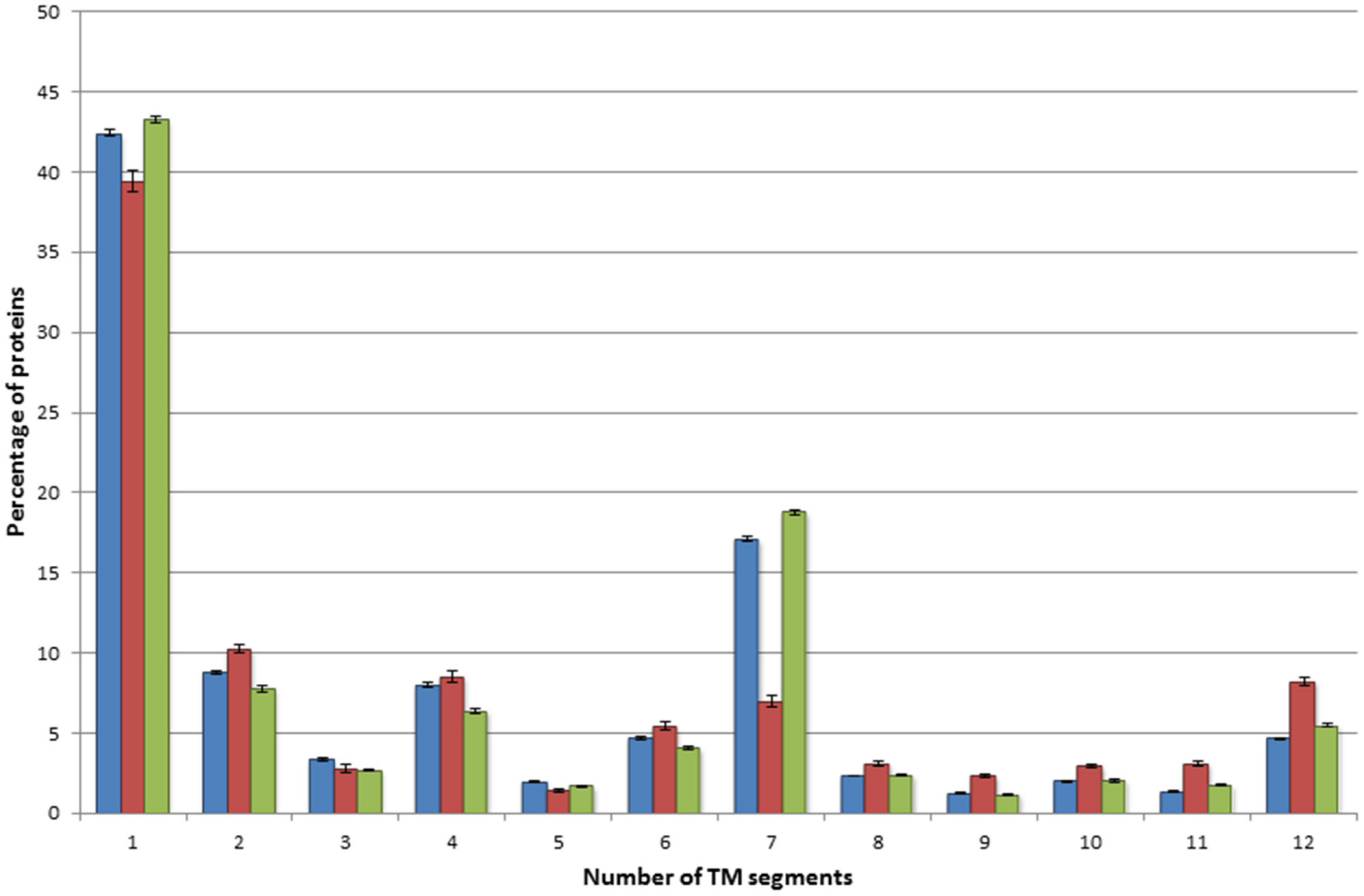
Distribution of genetic variations across transmembrane proteins. Blue bars represent proteins from the Human Transmembrane Proteome database, red bars represent TMPs containing disease-associated mutation(s), green bars represent TMPs containing polymorphism(s). TMPs with more than 12 TM segments were not included in the figure because of the low number of those proteins.

The distribution of the number of TM regions in TMPs containing polymorphisms is in good correlation with the distribution of TM regions in the whole HTP set (Fig 1). For example, the percentage of 7 TM TMPs with polymorphisms is similar to that of 7 TM TMPs in the HTP set (Fig 1). TMPs containing polymorphisms include 597 UniProt accesion IDs of TMPs containing 7 TM regions, which are unambiguously mapped to 591 unique Entrez Gene IDs using WebGestalt. About 50% of these (298) are classified as olfactory receptors by the KEGG enrichment analysis. In view of the significant variability of olfactory receptors within the human population [21,22], the high polymorphism rate in these TMPs is not surprising. As mentioned above, disease associated mutations accumulate in fewer proteins than polymorphisms, and the distribution of the number of TM segments in these two sets also shows significant differences. The relative decrease of the 7TM protein category within the disease associated mutation containing TMPs is the most noticeable difference, while there is a minor increase in other TM categories (with the exception of 1TM proteins, Fig 1). TMPs containing 10 or 12 TM regions are especially relevant since these classes mostly contain ion transport proteins with essential functions in the cell (as opposed to 7TMH TMPs determining the sense of smell). This analysis clearly shows the different occurrences of the polymorphisms and disease associated mutations in different types of TMPs.

### Comparing the distribution of amino acid substitutions between polymorphisms and disease associated mutations within distinct topology segments of transmembrane proteins

**Fig 2:**
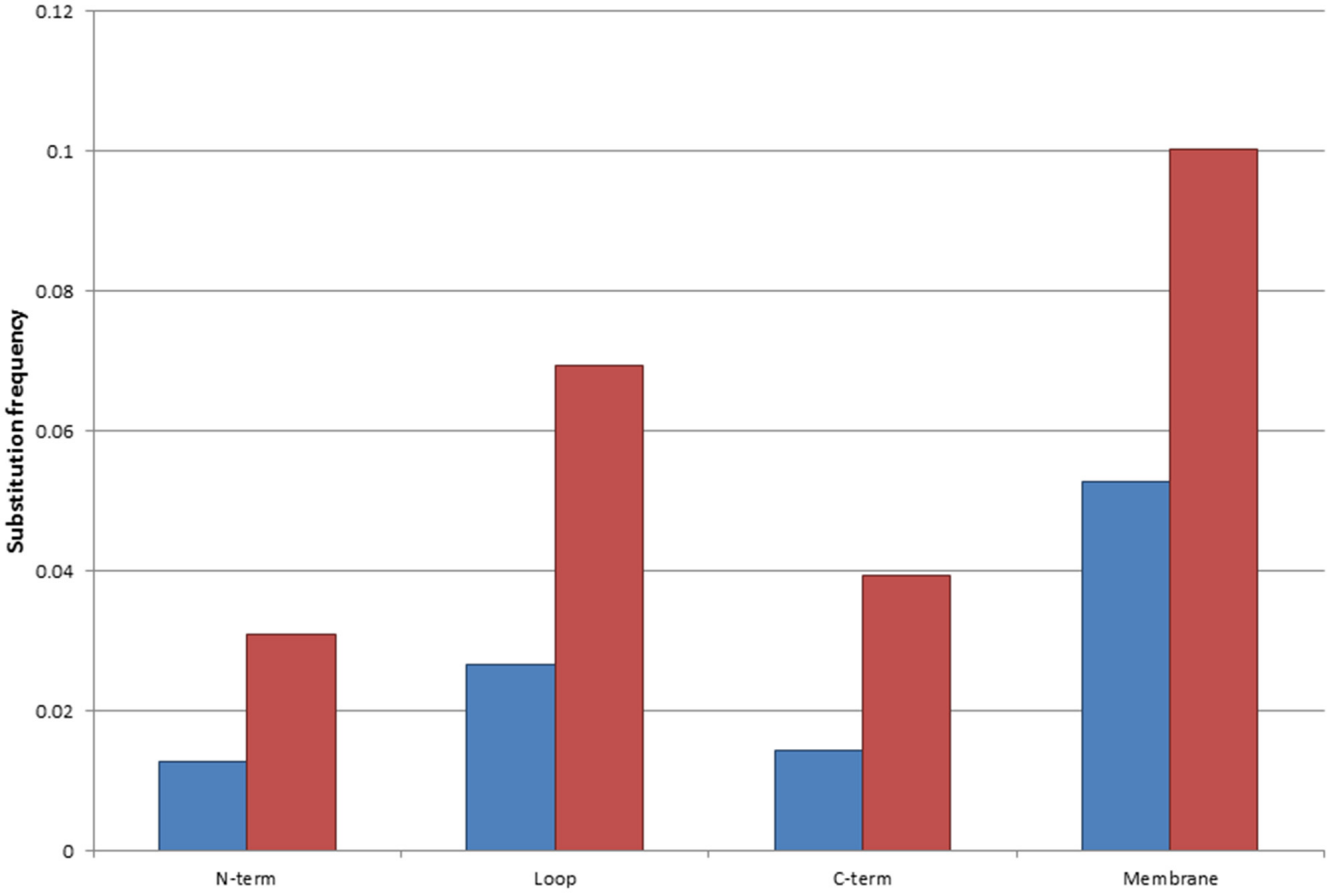
Substitution frequency within distinct regions of the TMP(s). Blue bars represent disease-associated mutations, red bars represent polymorphisms. Values were counted within the distinct regions of the proteins, and normalized to the length of each segment.

Experimental determination of TMP structures has proved rather challenging [23–25]. Therefore, bioinformatics tools play an important role in the prediction and investigation of structural information. The topology of TMPs may be considered as a “low resolution structure”, which determines the position of amino acid residues relative to the membrane plane. TMPs contain intracellular, transmembrane and extracytosolic segments (a more detailed description can be found on the http://topdb.enzim.hu web page [26]). We examined the distribution of the frequency of polymorphisms and disease associated mutations within these distinct segments by normalizing the occurrences of variations to the length of the respective topological segments. Interestingly, the highest frequency of polymorphisms and disease associated mutations are found in the transmembrane regions (Fig 2). In the case of polymorphisms non-polar to non-polar mutations are the most frequent (Table 1), whereas disease associated variations are typically non-polar to charged, and non-polar to non-polar mutations (Table 2). It is well known that the α-helical structures of the TMPs consist of mostly non-polar amino acid residues, which play a fundamental role in the formation of the hydrophobic TM segments and their interaction with the lipid bilayer. Since these types of interactions are not specific, polymorphisms are frequently tolerated as long as the resulting amino acid remains non-polar. In the case of disease associated variations, non-polar to charged amino acid changes provide polarity to the TM region, which disrupt the folding of the protein [16–18]. The most common polymorphisms in the TM regions result in valine to leucine, isoleucine to valine, alanine to threonine and phenylalanine to leucine substitutions (Table 3). In contrast to these polymorphic variations, in the case of disease associated mutations, the two most abundant changes are the glycine to arginine and the leucine to proline mutations (Table 4). These substitutions can be easily explained in view of the standard genetic code table, which shows that a single nucleotide change is sufficient to change at least four codons to induce either glycine to arginine or leucine to proline change.

**Table 1:** Relative frequency of the various type of amino acid substitutions within the TM regions associated with polymorphisms.

%	charged	non-polar	Polar
Charged	1,67±0,1	2,06±0,09	1,29±0,07
non-polar	4,22±0,13	53,83±0,41	18,35±0,35
Polar	2,65±0,1	13,09±0,23	2,84±0,11

**Table 2:** Relative frequency of the various type of amino acid substitutions within the TM regions associated with diseases.

%	Charged	non-polar	polar
charged	3,68±0,2	3,86±0,15	3,94±0,12
non-polar	22,61±0,42	33,09±0,24	12,1±0,3
Polar	5,85±0,18	11,35±0,22	3,5±0,11

**Table 3:** Relative frequency of amino acid substitutions. Mutated amino acids are shown in rows; mutant amino acids are shown in columns associated with polymorphisms.

Polymorphisms																				
%	A	R	N	D	C	Q	E	G	H	I	L	K	M	F	P	S	T	W	W	V
A	0	0	0	0,12	0	0	0,31	0,43	0	0	0	0	0	0	0,74	1,05	5,54	0	0	4,06
R	0	0	0	0	0,49	0,55	0	0,25	0,37	0	0,18	0,06	0,12	0	0,18	0,06	0	0,31	0	0
N	0	0	0	0,25	0	0	0	0	0,06	0,25	0	0,31	0	0	0	0,62	0,06	0	0	0
D	0,06	0	0,25	0	0	0	0,12	0,12	0,12	0	0	0	0	0	0	0	0	0	0,06	0
C	0	0,55	0	0	0	0	0	0,31	0	0	0	0	0	0,12	0	0,43	0	0,43	0,55	0
Q	0	0,37	0	0	0	0	0	0	0,18	0	0	0	0	0	0	0	0	0	0	0
E	0,12	0	0	0,06	0	0,06	0	0	0	0	0	0,06	0	0	0	0	0	0	0	0,06
G	0,55	0,74	0	0,43	0,43	0	0,37	0	0	0	0	0	0	0	0	1,29	0	0,12	0	0,43
H	0	0,62	0,12	0	0	0,06	0	0	0	0	0	0	0	0	0	0	0	0	0	0
I	0	0	0,43	0	0	0	0	0	0	0	0,68	0	0,92	0,55	0	0,12	3,69	0	0	6,09
L	0	0,37	0	0	0	0,31	0	0	0,25	1,05	0	0	0,8	3,26	1,6	0,55	0	0,12	0	2,34
K	0	0,25	0,12	0	0	0	0	0	0	0	0	0	0,12	0	0	0	0	0	0	0
M	0	0,06	0	0	0	0	0	0	0	1,05	0,86	0,18	0	0	0	0	1,78	0	0	2,71
F	0	0	0	0	0,55	0	0	0	0	0,06	4,49	0	0	0	0	1,23	0	0	0,31	0,62
P	0,06	0,18	0	0	0	0,06	0	0	0,06	0	0,8	0	0	0	0	0,86	0,12	0	0	0
S	0,74	0,18	0,55	0	0,55	0	0	0,98	0	0,25	1,11	0	0	0,62	0,8	0	0,62	0,06	0,12	0
T	2,71	0,25	0,18	0	0	0	0	0	0	1,48	0	0,31	1,66	0	0,43	0,55	0	0	0	0
W	0	0,18	0	0	0,37	0	0	0,06	0	0	0,12	0	0	0	0	0,12	0	0	0	0
Y	0	0	0,06	0	1,35	0	0	0	0,74	0	0	0	0	0,18	0	0,18	0	0	0	0
V	1,78	0	0	0,18	0	0	0,25	0,8	0	7,01	2,28	0	4,37	0,55	0	0	0	0	0	0

**Table 4:** Relative frequency of amino acid substitutions. Mutated amino acids are shown in rows; mutant amino acids are shown in columns associated with diseases.

Disease associated mutations																				
%	A	R	N	D	C	Q	E	G	H	I	L	K	M	F	P	S	T	W	W	V
A	0	0	0	1,39	0	0	0,98	0,46	0	0,05	0	0	0	0	1,19	0,26	2,53	0	0	2,68
R	0	0	0	0	1,08	1,14	0,05	0,15	1,39	0	0,36	0	0,05	0	0,36	0,26	0	0,83	0	0
N	0	0	0	0,62	0	0	0	0	0,1	0,36	0	0,72	0	0	0	0,77	0,15	0	0,21	0
D	0	0	0,88	0	0	0	0,15	0,26	0,15	0	0	0	0	0	0	0	0	0	0,67	0,21
C	0	1,65	0	0	0	0	0	0,15	0	0	0	0	0	0,15	0	0,21	0	0,26	0,98	0
Q	0	0,36	0	0	0	0	0,05	0	0,15	0	0,1	0,15	0	0	0,21	0	0	0	0	0
E	0	0	0	0,1	0	0,05	0	0,36	0	0	0	0,93	0	0	0	0	0	0	0	0
G	0,72	6,91	0	2,63	0,41	0	1,7	0	0	0	0,05	0,05	0	0	0	1,7	0	0,26	0	1,91
H	0	0,77	0,05	0,05	0	0,21	0	0	0	0	0,05	0	0	0	0,21	0	0	0	0,46	0
I	0	0,21	0,83	0	0	0	0	0	0	0	0,26	0,31	0,41	0,72	0	0,31	0,62	0	0	0,67
L	0	1,81	0	0	0	0,26	0	0	0,46	0,21	0	0	0,15	1,14	5,47	0,46	0	0,26	0	0,88
K	0	0	0,1	0	0	0	0,1	0	0	0	0	0	0	0	0	0	0,1	0	0	0
M	0	0,67	0	0	0	0	0	0	0	1,03	0,31	0,83	0	0	0	0	0,98	0	0	1,08
F	0	0	0	0	0,46	0	0	0	0	0,1	1,81	0	0	0	0	1,14	0,05	0	0,05	0,36
P	0,21	0,72	0	0	0	0,15	0	0	0,1	0	1,81	0	0	0	0	1,08	0,1	0	0	0
S	0	1,34	0,67	0	0,36	0	0	0,1	0	0,57	1,29	0	0	1,55	1,14	0	0,1	0,36	0,57	0
T	0,46	1,14	0,41	0	0	0	0	0	0	0,98	0	0,46	1,65	0	0,52	0,15	0	0	0	0
W	0	1,19	0	0	0,72	0	0	0,05	0	0	0,21	0	0	0	0	0,41	0	0	0	0
Y	0	0	0,1	0,15	1,6	0	0	0	0,62	0	0	0	0	0	0	0,36	0	0	0	0
V	0,72	0	0	0,62	0	0	0,41	0,31	0	1,7	0,57	0	2,06	1,03	0	0	0	0	0	0

We counted the amino acid substitutions for each topological region (polymorphisms and disease associated mutations) and compared the distributions to random sampling (S2 and S3 Files). Variations caused by the mutation of arginine residues are overrepresented in all topological segments, except within the transmembrane region where these amino acids are uncommon. This high mutability is due to the naturally occurring deamination of CpG dinucleotides in coding sequences. Polymorphisms within the TM regions are mainly apolar-to-apolar changes as shown in Table 1. These changes are highly overrepresented (S2 File) and symmetrical (e.g. valine to isoleucine and isoleucine to valine changes). In the case of disease associated mutations two prominent signatures can be identified (S3 File). First, cysteine residues are highly mutated in the extra-cytosolic region of TMPs, which can destabilize protein structure by altering disulphide bonds. The other change results in a glycine to arginine substitution within the TM region, which is characterized in more detail below.

### Characterizing the glycine to arginine and leucine to proline mutations

**Fig 3:**
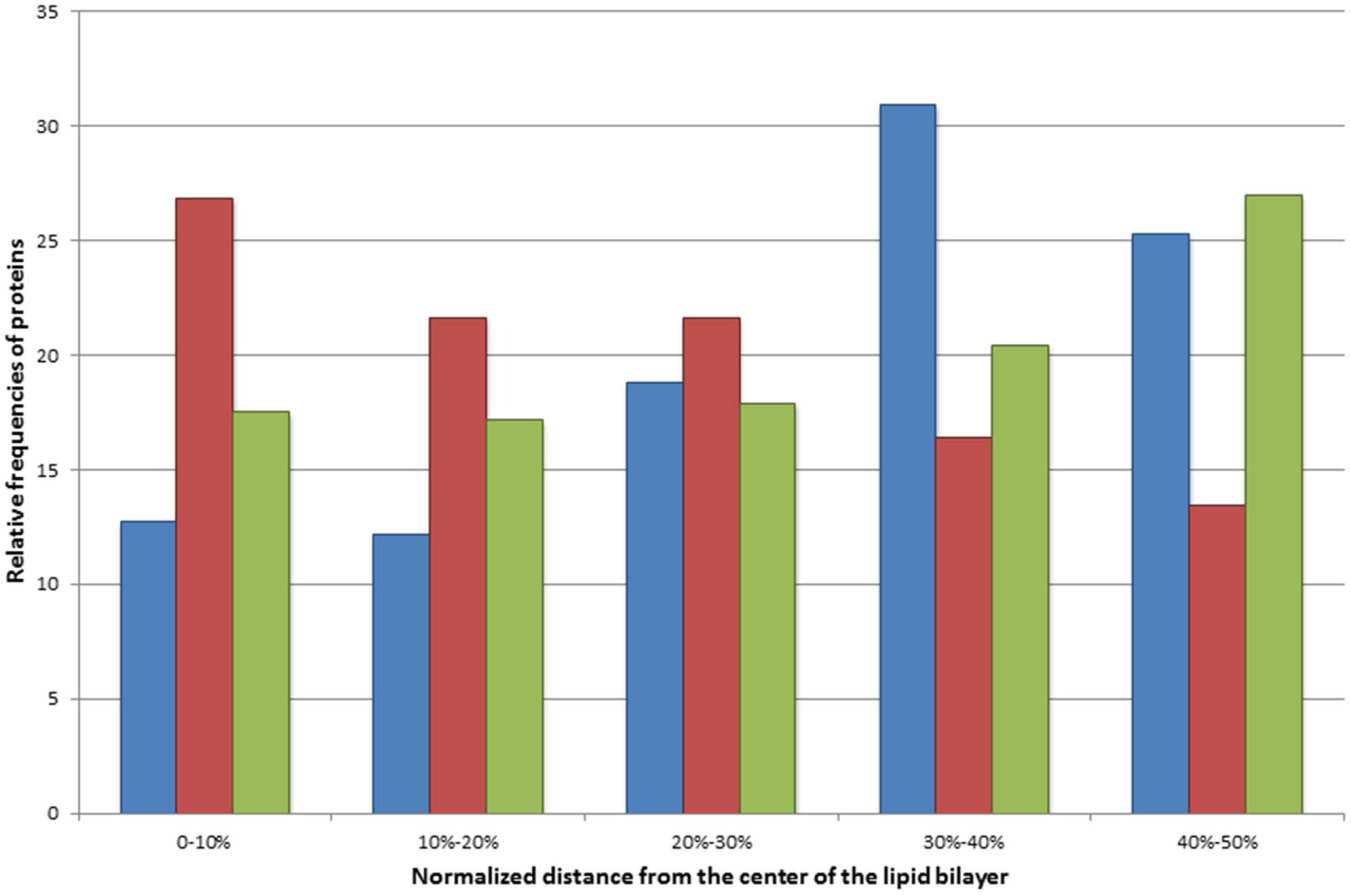
Relative frequencies of mutations in the membrane regions of transmembrane proteins. The distance was measured in the sequence from the most central amino acid in the transmembrane region and was normalized by the length (in residue) of the TM regions. Blue bars represent naturally occurring arginine residues within the TM region. Red bars represent the glycine to arginine disease-associated mutations within the TM region. Green bars represent all polymorphisms found within the TM region.

**Fig 4:**
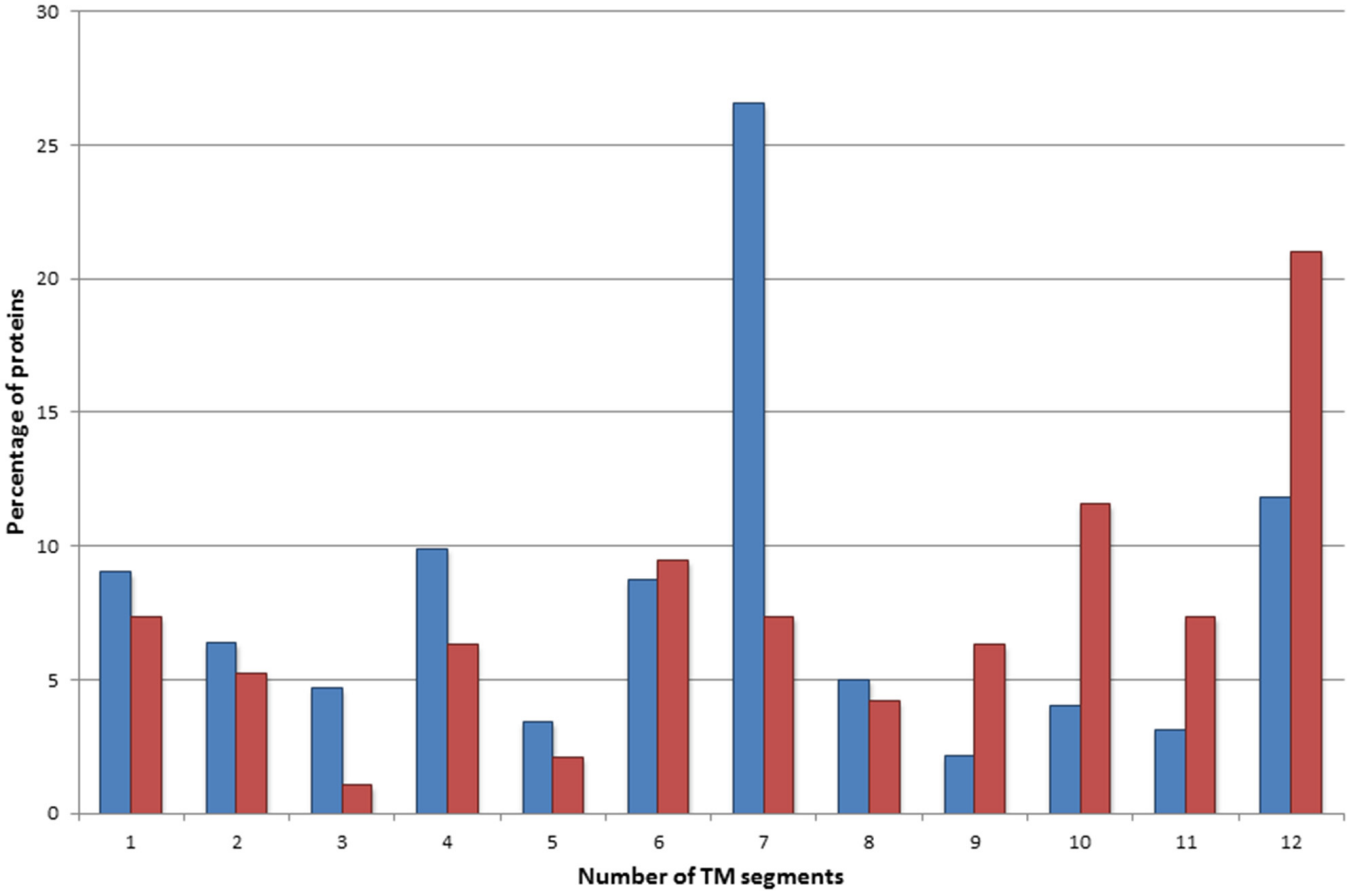
Distribution of TMPs containing naturally occurring arginine amino acids and disease associated glycine to arginine mutations within the TM region. Blue bars represent those proteins which contain arginine residue(s) in the TM segment. Red bars represent those proteins which contain disease-associated glycine to arginine mutation(s) within the TM segment.

To assess the relevance of glycine to arginine mutations, their occurrence in the transmembrane segments was compared with that of the naturally occurring arginines and polymorphisms resulting in arginine within the transmembrane regions (Fig 3). The disease associated glycine to arginine mutations were identified mostly in the center of the lipid bilayer, contrary to the naturally occurring arginines and polymorphisms, which are common towards the polar head groups of the lipid bilayer, in line with the notion that the extra charge in the depth of the lipid bilayer leads to a more severe deviation in the structure and function of the TMPs. Interestingly, naturally occurring arginine residues within TM segments can be found almost exclusively within TMPs containing 7 TM regions. Glycine to arginine mutations are frequently present in TMPs containing 10 and 12 TM regions (Fig 4). This observation suggests that arginines naturally occurring in TMPs containing 7 TM regions (mostly G protein-coupled receptors) have a dedicated role, in comparison to ion channels or ion transport proteins where similar variations result in a disease phenotype.

The leucine to proline variations are non-polar to non-polar substitutions, hence these are not providing extra charge into the TM region by the amino acid side chains. However, proline can cause major disturbances by disrupting the hydrogen bridge system of the α-helices and exposing a hydrogen bridge acceptor, which provides a partial extra charge within the lipid bilayer. Therefore, it is not surprising that the enrichment analysis revealed that TMPs containing this type of mutations are frequent among the10 and 12 TM TMPs with ion transport function. A bootstrap method performed to estimate the significance of the observed count of mutations revealed that glycine to arginine and leucine to proline mutations located in transmembrane segments significantly differ from each other. While the glycine to arginine mutation was found to be highly significant, the high count of leucine to proline mutations is the result of chance (see S2 and S3 Files).

Additionally we determined a “predictive value” for those mutations which occur frequently (more than hundred times) within the TM region (S4 File). This analysis clearly shows that the glycine to arginine changes in the membrane regions are the highest occurring disease causing mutations.

**Fig 5:**
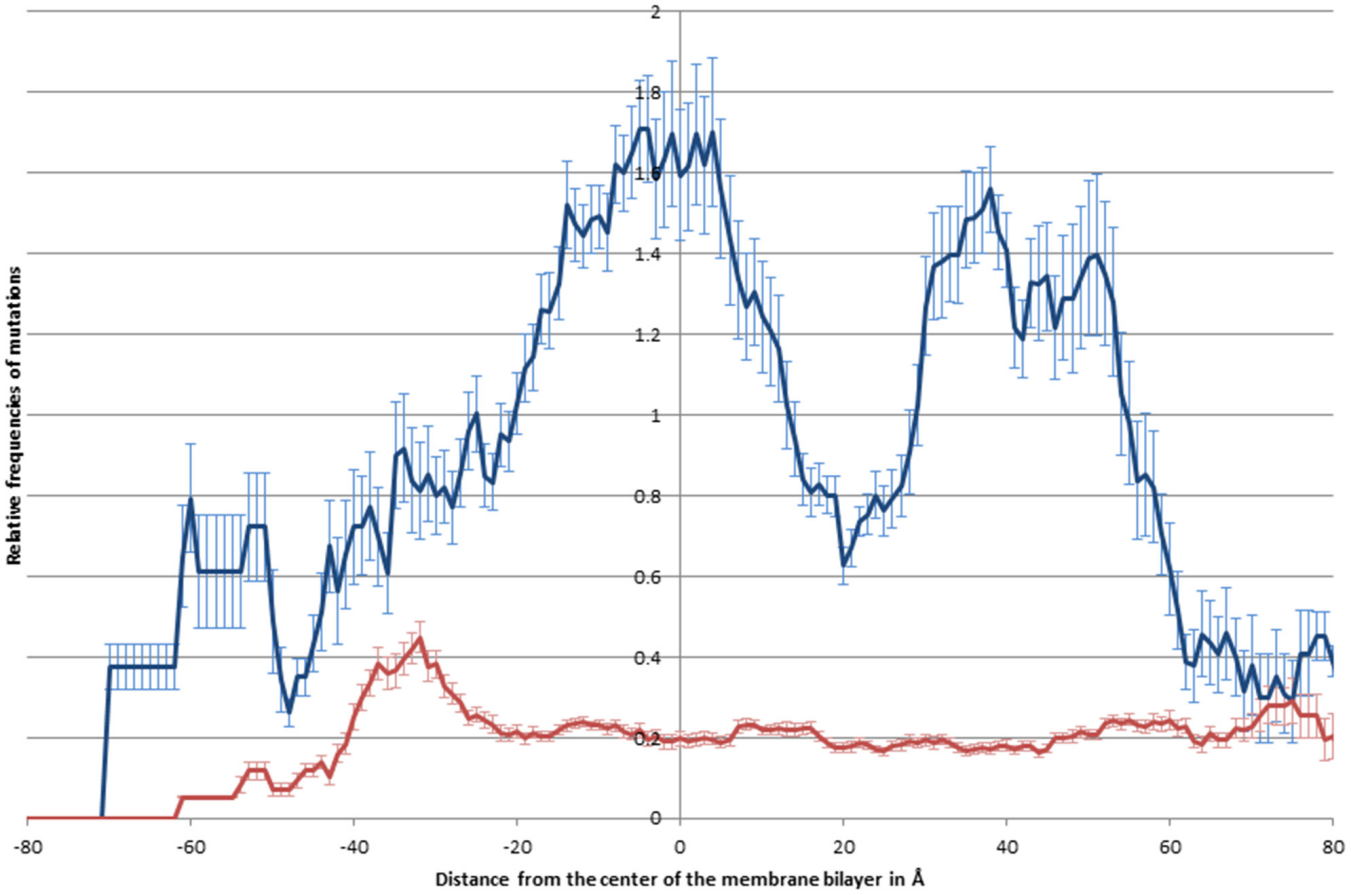
Distribution of relative frequencies of mutations along the z-axis. The relative frequencies of disease causing mutations (blue) and polymorphisms (red) are shown along the z-axis See Materials and Methods for details.

The relative frequencies of polymorphisms and disease associated mutations were further characterized by mapping their positions on available 3D structures. The distribution of the variations was evaluated along the z-axis (Fig 5). While the distribution of polymorphisms show no significant changes along the z-axis, the distribution of the relative frequencies of disease associated mutations are more abundant in the middle of the double lipid layer, similarly to the distribution of glycine to arginine variations. However, our analysis also revealed that the disease associated mutations have two other maximums close to the head groups of the lipid molecules in the cytosolic membrane leaflet (~38Å) and in the cytosolic water soluble part of TMPs (~50Å) (Fig 5).

## Discussion

In this study we combined information obtained from the Human Transmembrane Proteome and UniProt databases to analyze the characteristics of naturally occurring missense genetic mutations in human TMPs. In particular, our aim was to compare the distribution of polymorphic and disease associated variations within distinct protein segments of several TMP classes.

Despite the similar distribution of polymorphisms and disease associated mutations across topological regions, the composition of the amino acid changes were found to be different. In the case of polymorphic variations, changes within the TM regions most frequently retain the apolar nature of the amino acids, whereas disease associated mutations result in characteristic apolar to charged or apolar to apolar changes. The amino acid residue substitution matrix of the human transmembrane proteome reveals that the glycine to arginine changes are primarily responsible for this phenomenon (Table 4). This makes sense, since the arginine residue provides an extra charge in the lipid bilayer, which may dramatically alter the structure and function of TMPs. Although arginines occur rarely in the membrane spanning regions of TMP, there are examples of naturally occurring arginine residues within the TM segments of TMPs. We find that arginines introduced into the membrane spanning segment by the glycine to arginine amino acid variations accumulate primarily within the interior of the lipid bilayer.

Glycine to arginine changes are more likely to be relevant when the variation affects a conserved position. For example, the *p*.*Gly833Arg* mutation causes a 78% reduction in the expression of the GRIA3 protein [27]. There are several literature reports that confirm the pathological relevance of glycine to arginine changes within the membrane regions of TMPs. For example, the *p*.*Gly380Arg* mutation in the FGFR3 protein is believed to be responsible for achondroplasia, [28]; the *p*.*Gly185Arg* change in the NRAMP2 protein results in microcytic aneamia in humans [29]; the *p*.*Gly796Arg* variation in the Band3 protein causes hereditary stomatocytosis [30]. Interestingly, this latter mutation is not listed in the major mutational databases including UniProt, Ensembl, or dbSNP. Since the UniProt classification of missense mutations represents the probability of disease association (based on theoretical considerations; see the description at the website: http://www.uniprot.org/docs/humsavar), it cannot be used for clinical or diagnostic use. Surprisingly, our analysis identified 12 glycine to arginine variations in the membrane spanning segments of TMPs that are nevertheless annotated as polymorphisms in the UniProt database (see S6 File). Analysis of the literature revealed that three of these variations result in altered phenotypes (two being disease causing, the rs34059508 and rs36209700 variants in the SLC22A1 [31] and ABCG8 [32] proteins respectively), suggesting that these variations are misclassified in the UniProt. The six remaining variations occur rarely in the human population, which may explain why a phenotype has not been identified. In fact, the erroneous annotation of sequence variations may have clinically relevant consequences. For example, a study found that 87% of patients with fibrodysplasia ossificans progressiva (FOP) were originally misdiagnosed [33], and the link of an atypical variation to the disease was only revealed by whole exome sequencing [34].

The identification of relevant mutations in whole genomes has been linked to finding a needle in a haystack [35]. Today, ongoing efforts in the United States [36], Canada (the FORGE project [37,38]) and in the UK (The Rare Diseases Genomes Project of Genomics England) are sequencing thousands of genomes to identify genes that responsible for rare Mendelian diseases. In this era of genomic data deluge, when sequencing machines generate more data than researchers can analyze, the evaluation of the relevance of sequence variations is increasingly important. We suggest that low resolution structural information, such as the transmembrane topology of TMPs provides an important contribution to the evaluation of the functional relevance of genetic variations. The analysis of sequence variations in the context of topological information should help the identification of functionally relevant mutations that are more likely to be associated with a clinically relevant phenotype.

## Materials and Methods

### Databases

Human genetic variation data (polymorphisms and disease mutations) was obtained from the UniProt database (version 2014_10) (http://www.uniprot.org/docs/humsavar.txt, date of release: 29-Oct-2014) [39]. This release contains 69978 naturally occurring human genetic missense variations, among these 25310 are disease associated, 38030 are polymorphisms, and 6638 are unclassified respectively.

Identifiers of human non-TMPs as well as the topology information for the human TMPs were imported from the HTP database (http://htp.enzim.hu/data/database/sets/htp_all_uniprot13_03.xml) [19]. The downloaded version of HTP database consists of 14586 non-TM and 4998 TMPs.

### Analyzing the human transmembrane protein variations

Genetic variations for the 4998 human α-helical TMPs as well as for the 14586 non-TMPs were imported from the UniProt database. The unclassified mutations were excluded from the analysis. All topology and variation data for TMPs were converted to the standard Bed format [40], using the UniProt ID and the position of the variant and were inserted before the original columns of the annotation. Ambiguous variations associated with multiple diseases were removed. In the case of multi-pass membrane proteins, we distinguished the terminals (regions before the first and after the last TM segments) from the loop regions and added this information to the converted files (e.g., N-terminal, Loop, C-terminal). The overlaps between the variations and the different segments of topology were determined by the intersectBed program, with the option—wo, from the Bedtools software package version v2.17.0 [41]. A step by step description of these preanalytical steps can be found in the S5 File. Using the original UniProt annotation of these variations within the different topological sites, the exact amino acid substitutions and the grouping by the polar/non-polar/charged protein property were counted by a Perl script. We counted asparagine, glutamine, serine, threonine and tyrosine as polar residues; alanine, cysteine, glycine, isoleucine, leucine, metionine, phenylalanin, proline, tryptophan and valine as non-polar residues; arginine, aspartic acid, glutamic acid, histidine, and lysine as charged residues. To estimate the standard deviation of the distribution of the various substitution types, we applied a bootstrap method by selecting the 90% of variations from the disease associated and polymorphism groups by chance for ten times, and the mean and standard deviation values from the ten cases were calculated. The significance of the observed amino acid substitution matrix for the different topological sites was tested. In the case of the three topological sites (inside, membrane, outside) positions were randomly chosen for every observed mutant sites from amino acid sequences located within those regions. The observed substitution rate was used to construct a random substitution matrix for the amino acid changes. This method was applied a hundred times and the average and standard deviation values were determined to all substitutions, then the significance of the observed values was examined. The distance of the glycine to arginine variations from the center of the transmembrane region was computed by a Perl script. The enrichment analyses were determined by the WebGestalt web service [42], using the default options, and the hsapiens__entrezgene_protein-coding reference set. The EMBOSS software package version 6.3.1 was used to manipulate the raw protein sequences, and to obtain the information of protein sequences [43]. The perl scripts can be downloaded from the following web page: http://mbk.enzim.ttk.mta.hu/TMmutations.

For the investigation of the distribution of mutations in the 3D structures of TMPs, the polymorphisms and disease associated mutations were mapped onto the 3D structures of TMPs; the membrane normal was parallel with the z-axis and the zero point was in the middle of the double lipid layer. The information for the necessary rotation was taken from the PDBTM database [44]. The proteins were cut into 1Å wide slices parallel to the membrane plane, and the number of polymorphisms and disease associated mutations as well as the number of all residues were summed for each TMP having homologous structure in PDBTM database. The relative frequencies of mutations were calculated by dividing the sums by the sum of all residues in each slice.

## Supporting Information

S1 File Topological annotation of polymorphisms and disease associated mutations. Human protein variation data was downloaded from the UniProt database release 29-Oct-2014. The topology information was obtained from the HTP database version 1.0. The creation of this file is described in the Materials and Methods section of this paper. The abbreviations for the Topology_type are I (inside), M (membrane), O (outside).
(XLSX)

S2 File The significance of the different amino acid substitutions of polymorphisms within distinct topological sites. Tables represent the observed (OBS) values, the average values of random sampling of hundred times (AVG), the standard deviation of the random sampling (STD) and the ratio of the observed minus average and standard deviation values ((OBS-AVG)/STD).
(XLSX)

S3 File The significance of the different amino acid substitutions of disease associated mutations within distinct topological sites. Tables represent the observed (OBS) values, the average values of random sampling of hundred times (AVG), the standard deviation of the random sampling (STD) and the ratio of the observed minus average and standard deviation values ((OBS-AVG)/STD).
(XLSX)

S4 File Occurrences of polymorphisms and disease associated mutations in the TM region and the “predictive value” table. Tables represent the counts of occurrences of the specific amino acid changes within the TM region in the case of disease associated mutations and polymorphisms. Additionally there is a worksheet which shows the summarized counts of the disease associated mutations and polymorphisms. The fourth worksheet contains the “predictive value” which was counted from the number of disease associated mutations divided by the summarized value of the specific amino acid changes, when the summarized value is greater than 100.
(XLSX)

S5 File Step by step description of preanalysis. A detailed description of the steps which are necessary to reproduce our analysis.
(DOCX)

S6 File Phenotypic variance and minor allele frequency of the glycine to arginine polymorphisms within the UniProt database. Glycine to arginine changes annotated as polymorphisms in the UniProt database. The table contains the gene name, the UniProt accession of the protein, the UniProt variant identifier, the rsID of the polymorphism (if applicable), phenotypic variance with the PubMed identifier and the minor allele frequency (if applicable) from the Ensembl database (version 83, release: December 2015).
(XLSX)
