# Supplementary material for: Characterization of Disease-Associated Mutations in Human Transmembrane Proteins
Source: PLoS One. 2016 Mar 17;11(3):e0151760. doi: 10.1371/journal.pone.0151760 (PMC4795776; doi:10.1371/journal.pone.0151760)
Supplement: S5 File — A detailed description of the steps which are necessary to reproduce our analysis. (DOCX) [file pone.0151760.s005.docx]

**Step1**. Download the humsavar.txt from UniProt <http://www.uniprot.org/docs/humsavar>

We used the database version 2014_10 which was released on 29-Oct-2014.

**Step2**. Filter out “Unclassified variants”

**Step3**. Lines containing the annotation of “Polymorphism” will be those mutations which are not disease associated.

**Step4**. Lines containing the annotation of “Disease” will be those mutations which are disease associated. But the annotation can be redundant for some of the mutations, and this redundancy must be removed, so we kept only one of these.

For example:

| Main gene name | Swiss-Prot AC | FTId | AA change | Type of Variant | dbSNP | Disease name |
| --- | --- | --- | --- | --- | --- | --- |
| CACNA1A | O00555 | VAR_001492 | p.Thr666Met | Disease | rs121908212 | Episodic ataxia 2 (EA2) [MIM:108500] |
| CACNA1A | O00555 | VAR_001492 | p.Thr666Met | Disease | rs121908212 | Migraine, familial hemiplegic, 1 (FHM1) [MIM:141500] |

**Step5**. The polymorphisms and disease associated files can be formatted to format similar to “BED” format (a basic coordinate system format). In this case the order of the columns will be changed from the humsavar format (Main gene name, Swiss-Prot AC, FTId, AA change, Type of variant, dbSNP, Disease name) to the “BED”-like format (Swiss-Prot AC, Variation Start, Variation End, Main gene name, FTId, AA change). Using the “BED”-like format intersection of different features with coordinates can be easily computed. To this the bedtools program needs coordinates with columns start and end. The start and end of the mutation can be easily determined using the information from the AA change column from the humsavar.txt. Example: Thr666Met can be converted to start 665 and end 666 respectively.

**Step6**. Using the topology information obtaining from the HTP, a similar process can be done to convert the xml file to “BED”-like format, using the UniProt protein accession ID, and the topology information to determine the start and end of the region within the protein sequence.

The HTP version which was used can be accessed under this link:

http://htp.enzim.hu/data/database/sets/htp_all_uniprot13_03.xml

**Step7**. Using the Bedtools: intersectBed –a Polymorphism/Disease-associated-mutations.bed –b HTP-topology.bed –wo > Polymorphism/Disease-associated-mutations_vs_HTP-topology.bed (these result files can be found in the Supporting_file_S1.xlsx and http://mbk.enzim.ttk.mta.hu/TMmutations/data folder with the name of polymorphism.txt and disease.txt)

**Step8**. Use the run.sh (downloaded from http://mbk.enzim.ttk.mta.hu/TMmutations/run.sh) in the folder to get the results in the article.
